# Supplementary material for: Getting to GRIPS with MR-Egger: Modelling directional pleiotropy independently of allele coding
Source: PLoS Genet. 2025 Dec 30;21(12):e1011967. doi: 10.1371/journal.pgen.1011967 (PMC12753088; doi:10.1371/journal.pgen.1011967)
Supplement: Attachment — Submitted filename: Dudbridge.pdf [file pgen.1011967.s003.pdf]

Review of "Getting to GRIPS with MR-Egger: modelling directional pleiotropy independently of allele coding"

The validity of the MR-Egger regression relies on the InSIDE assumption. However, this assumption is specific to the coding of the instrument SNPs. This is problematic because there are many equivalent ways to code a set of SNPs. This manuscript addresses this problem by proposing a new regression model that has the Genotype Recoding Invariance Property (GRIP).

While this work is on an important topic, there are several issues that dampen my enthusiasm.

- Like many other MR methods, this research implicitly assumes $\{\hat{\gamma}_j\}$ are mutually independent, so are $\{\hat{\Gamma}_j\}$. This should be acknowledged.
- The proposed model is

$$\hat{\Gamma}_j \hat{\gamma}_j = \beta \hat{\gamma}_j^2 + \alpha_j \hat{\gamma}_j + \epsilon_{Yj} \hat{\gamma}_j.$$

  There is no discussion of the following important issues:

  - The modeling part $\beta \hat{\gamma}_j^2 + \alpha_j \hat{\gamma}_j$ is correlated with the error $\epsilon_{Yj} \hat{\gamma}_j$. Will this lead to bias in β estimate?
  - It is not clear how β is estimated. In the zero-intercept case, the authors indicated that weighted least squares is used. If so, as the variance of $\epsilon_{Yj} \hat{\gamma}_j$ is $\hat{\gamma}_j^2 \sigma_{Yj}^2$, shouldn't a weighted least squares on the proposed model be identical to a weighted least square on the following original model?

$$\hat{\Gamma}_j = \beta \hat{\gamma}_j + \alpha_j + \epsilon_{Yj}.$$

Overall, this work has large room for improvement in at least the theoretical part.
