# Supplementary material for: Getting to GRIPS with MR-Egger: Modelling directional pleiotropy independently of allele coding
Source: PLoS Genet. 2025 Dec 30;21(12):e1011967. doi: 10.1371/journal.pgen.1011967 (PMC12753088; doi:10.1371/journal.pgen.1011967)
Supplement: Attachment — Submitted filename: main.pdf [file pgen.1011967.s004.pdf]

Paper Title: *Getting to GRIPS with MR-Egger: modelling directional pleiotropy independently of allele coding*
Recommendation: Major revision

1. Summary

The manuscript introduces a new identifying assumption, "Variance Independent of Covariance Explained" (VICE), and an accompanying estimator, MR-GRIP, which is invariant to allele coding and offers clearer practical interpretation than MR-Egger. By modifying the causal model from allele orientation, the authors overcome the well-known fragility of the InSIDE assumption while preserving a valid test for directional pleiotropy. The exposition is coherent that the link between InSIDE and coding choice is carefully derived, the simulation design is thoughtfully constructed, and the real-data application convincingly demonstrates MR-GRIP's advantages over MR-Egger. Overall, the paper is clearly written, theoretically sound, and makes a meaningful contribution to Mendelian Randomization methodology. However, the paper is not clear on the new assumptions and the method is not well-explained.

2. Strengths

- **Methodological novelty.** Introduces the "Variance Independent of Covariance Explained" (VICE) assumption and the corresponding MR-GRIP estimator which invariant to allele coding, providing a new identification strategy for MR analyses affected by directional pleiotropy.
- **Insightful treatment of allele coding.** Provides a clear derivation showing how the InSIDE assumption depends on allele orientation and why recoding can jeopardise MR-Egger. This motivates VICE and underscores the practical value of MR-GRIP's coding-robust formulation.
- **Practical interpretability.** MR-GRIP uses coding-robust quantities (γ_j^2 and $\alpha_j \gamma_j$) eliminating a common source of user error in MR-Egger analyses. The intercept test remains meaningful when under null hypothesis $\alpha_0 = 0$.
- **Comprehensive simulation study.** Evaluates estimator bias, standard-error calibration, and power across a wide spectrum of pleiotropy patterns (balanced, directional, and correlated) and instrument strengths.
- **Convincing real-data application.** Demonstrates that MR-GRIP yields tighter confidence intervals and more consistent causal estimates than MR-Egger on a multi-trait lipid and cardiovascular-outcome dataset.

3. Detailed Comments: Weakness / Concerns

1. **Formal statement of VICE and scope of validity.** The manuscript gives an intuitive description of VICE but never spells it out in mathematical notation comparable to Equations (8)-(9) for the classical model. Readers are left unsure of the exact conditions under which MR-GRIP is identified. Please state VICE precisely (e.g., $\mathrm{Cov}(\alpha_j \gamma_j, \gamma_j^2) = 0$ or a conditional independence statement) and discuss whether it must hold for all SNPs or only in expectation over a mixture of valid and invalid instruments. Pages 11-12 outline why VICE is automatically

satisfied under InSIDE combined with balanced pleiotropy, and they hint at the resulting relationship between α_j and γ_j (which is also not clear). What is missing is a formal statement of the general conditions under which VICE is valid and how broad its applicability is.

2. **Limited theoretical contrast between VICE and InSIDE.** While the allele-coding argument is clear, the paper stops short of proving how VICE, InSIDE, and balanced pleiotropy intersect or diverge under directional pleiotropy. Based on the formal statement of VICE, the authors should provide a more detailed explanation of the relationship between VICE and InSIDE.

3. **Identifiability under weak instruments**. Section *Weak instruments* briefly notes that MR-GRIP can be debiased but the bias term under weak instrument asymptotics is not derived. Given that many real datasets encouters weak instruments, a paragraph quantifying how far the MR-GRIP slope drifts when strength of the instrument drops would be valuable Alternatively, provide a diagnostic or rule tailored to the γ_j^2 regression.

4. **Interpretability of the GRIP intercept.** The intercept estimates $E[\alpha_j \gamma_j]$, a quantity that is less intuitive than $E[\alpha_j]$ and receives minimal discussion. Concrete examples or practical thresholds would help practitioners judge its magnitude.

4. Additional Remarks

– Section *Introduction* paragraph 2: "This terminology ..." is not clear which it refers to.

– Page 11: "Although the converse assumption, independence of $\alpha_j \gamma_j$ and α_j^2, is not guaranteed, neither is it excluded by construction as it is for MR-Egger." This is not straightforward and should be explained.

– Page 14: "Estimands: the average causal effect of a unit shift intervention in X on the outcome Y, ie. $\beta = E[Y(X = x)] - E[Y(X = x - 1)]$, and the standard error of its estimator." The notation $Y(X = x)$ is not clear, is it using the potential outcome framework?

– Page 16: "The standard error of MR-GRIP is greater than IVW, WM and MBE, but lower than MR-Egger with all-positive coding. Overall, these results do not provide robust evidence for a causal effect of urate on CHD." The reason why MR-GRIP is more efficient than MR-Egger with all-positive coding is not clear. The same for the simulation results, the interpretation for Table 5 in Page 19.
