# Supplementary material for: Getting to GRIPS with MR-Egger: Modelling directional pleiotropy independently of allele coding
Source: PLoS Genet. 2025 Dec 30;21(12):e1011967. doi: 10.1371/journal.pgen.1011967 (PMC12753088; doi:10.1371/journal.pgen.1011967)
Supplement: Attachment — Submitted filename: Dudbridge2.pdf [file pgen.1011967.s006.pdf]

Review of "Getting to GRIPS with MR-Egger: modelling directional pleiotropy independently of allele coding"

I think this research and the original MR-Egger method are fundamentally flawed. I don't recommend the publication of this work.

The MR-Egger method is based on the theory developed by Kolesár et al. (2015. Identification and inference with many invalid instruments. *Journal of Business & Economic Statistics* 33 (4), 474–484). The InSIDE condition **incorrectly** interpreted the identification condition for the causal effect given in Kolesár et al. as the independence between $\{\alpha_j\}$ and $\{\gamma_j\}$ (with weights proportional to the inverse of $\sigma^2_{Y_j}$). The correct interpretation should be the independence between $\{\sum_j \alpha_j G_{ij}\}$ and $\{\sum_j \gamma_j G_{ij}\}$. In obvious matrix notations, this means the independence between $\boldsymbol{G\alpha}$ and $\boldsymbol{G\gamma}$. See also Eq. 11 of Kang et al. (2025, Identification and Inference with Invalid Instruments. *Annual Review of Statistics and Its Applications.* 12. 385-405).

Because of this incorrect interpretation, the InSIDE condition is misleading. The issue this paper trying to address is a result of this mistake. To identify the causal effect, the InSIDE condition can be replaced by the independence condition between $\boldsymbol{G\alpha}$ and $\boldsymbol{G\gamma}$. No matter how one codes the SNPs, the covariance between $\boldsymbol{G\alpha}$ and $\boldsymbol{G\gamma}$ is not affected.

Therefore, it is pointless to "improve" MR-Egger.

In addition, this manuscript contains a mistake: The estimated effect sizes $\hat{\gamma}_1, \hat{\gamma}_2, \ldots$ are not independent of each other even though the SNPs are in linkage equilibrium. See page 1478 for expression for $Cor2(\hat{\gamma}_j, \hat{\gamma}_k)$ in https://research-information.bris.ac.uk/ws/files/244353308/AOS1866.pdf (there is a typo in this expression: the multiplying factor 4 on the right hand side shouldn't be there).

Furthermore, in the derivation of the following relationship on page 12,

$$cov(\alpha\gamma, \gamma^2) = E(\alpha)cov(\gamma, \gamma^2),$$

it is implicitly assumed that $E(\alpha\gamma) = E(\alpha)E(\gamma)$. Is the InSIDE assumption needed for this to be true?
