# Supplementary material for: Getting to GRIPS with MR-Egger: Modelling directional pleiotropy independently of allele coding
Source: PLoS Genet. 2025 Dec 30;21(12):e1011967. doi: 10.1371/journal.pgen.1011967 (PMC12753088; doi:10.1371/journal.pgen.1011967)
Supplement: Attachment — Submitted filename: Review.pdf [file pgen.1011967.s007.pdf]

The manuscript now formally states and derives the VICE assumption (p.11), clarifies its relationship to InSIDE and provide an example of VICE (p.12), which directly addresses my earlier concern about the lack of a precise formulation, even if full justification still remains challenging in practice. But, there are still minor points:

- While the estimation framework is clearly presented, the authors did not provide an explicit formula for the variance of the MR-GRIP estimator. This seems straightforward to derive, but it would be important to include it for completeness, since variance estimation matters in practice for inference and implementation. And it can make the method more defensible.

- Although the intercept is treated as a nuisance parameter, the paper itself notes that its p-values are highly correlated with those of MR-Egger in the simulation. It would be interesting and informative to include simulation results illustrating the performance of the intercept test under MR-GRIP, even briefly, to support this point.
