# Supplementary material for: Getting to GRIPS with MR-Egger: Modelling directional pleiotropy independently of allele coding
Source: PLoS Genet. 2025 Dec 30;21(12):e1011967. doi: 10.1371/journal.pgen.1011967 (PMC12753088; doi:10.1371/journal.pgen.1011967)
Supplement: Attachment — Submitted filename: Response to reviews final.docx [file pgen.1011967.s008.docx]

# Response to reviews

## Getting to GRIPS with MR-Egger: modelling directional pleiotropy independently of allele coding

## Reviewer 1

The InSIDE condition incorrectly interpreted the identification condition for the causal effect given in Kolesár et al. as the independence between $\left\{ \alpha_{j} \right\}$ and $\left\{ \gamma_{j} \right\}$ … The correct interpretation should be the independence … between **Gα** and **Gγ** … Because of this incorrect interpretation, the InSIDE condition is misleading. The issue this paper trying to address is a result of this mistake. To identify the causal effect, the InSIDE condition can be replaced by the independence condition between Gα and Gγ. No matter how one codes the SNPs, the covariance between **Gα** and **Gγ** is not affected.

The Kolesár condition is associated with the k-class estimators in individual level data. It is not clear how it relates to summary-data estimators. If we replaced the InSIDE condition by the Kolesár condition, it would be unclear whether even the IVW estimator would be consistent.

MR-Egger was motivated by the meta-analysis formulation of the IVW estimator and is not a k-class estimator. Its InSIDE assumption does identify the causal effect, albeit under different scenarios to the Kolesár condition. Thus we disagree that the InSIDE assumption is a misinterpretation of the Kolesár condition: it was developed (by one of us) completely independently. It is a different condition, applying to a different estimator.

We do agree that the InSIDE condition is problematic in MR-Egger, which is the subject of our paper.

MR-GRIP also is a summary-data estimator with its own identification condition. It’s not hard to create a scenario where VICE holds but the Kolesár condition does not. Thus again, it is a different condition for a different estimator. At the very least, MR-GRIP widens the class of scenarios that can be identified with summary-level data.

… this manuscript contains a mistake: The estimated effect sizes  $\hat{\gamma}_{1}$, $\hat{\gamma}_{2}$ … are not independent of each other even though the SNPs are in linkage equilibrium. See page 1478 [*sic*] for expression for $cor^{2}(\hat{\gamma}_{1},\hat{\gamma}_{2}$) …

Thank you for pointing this out. We follow the argument on page 1748 of Zhao et al [8] that SNP effects are sufficiently small for this correlation to be ignored. We have reworded the text on page 5: “In line with other summary data methods we assume that the SNPs are independent (*ie* in linkage equilibrium) and have sufficiently small effects that, for $j\neq k$, the correlations between $\hat{\gamma}_{j}$ and $\hat{\gamma}_{k}$, and between $\hat{\Gamma}_{j}$ and $\hat{\Gamma}_{k}$, are negligible.”

… in the derivation of the following relationship on page 12, $cov\left( \alpha\gamma, \gamma^{2} \right)=E\left( \alpha\right)cov\left( \gamma,\gamma^{2} \right)$, it is implicitly assumed that $E\left( \alpha\gamma\right)=E\left( \alpha\right)E(\gamma)$. Is the InSIDE assumption needed for this to be true?

Yes, as was stated just above the derivation.

## Reviewer 2

1. For point 2, I remain confused by this statement “the graphical interpretation of IVW … only makes sense under all-positive coding”.
a. First, I suggest the authors clarify the motivation for introducing this discussion. Why is this statement conceptually or practically important to the argument being made?

We think it is important because many applied practitioners find the scatter plot helpful in understanding MR. We are describing an aspect of this plot that has not previously been recognised.

b. Second, as far as I understand, the slope of each point remains the same regardless of the coding choice, and therefore the average slope should also remain invariant. If this is the case, why would the graphical interpretation only make sense under all-positive coding?

The text is “the graphical interpretation of $\hat{\beta}_{IVW}$ as an average of individual slopes, and as a line of best fit through the data points, only makes sense under all-positive coding.” Looking at the black dots in figure 2b, the red line does not look like an average of the four lines joining the dots to the origin (this is the graphical interpretation), even if its slope is the average of the four slopes. Neither does the red line fit the black data points. However, those statements are appropriate for the hollow dots. We have modified the text to

“However, the graphical interpretation of $\hat{\beta}_{IVW}$ as an average of individual slopes, and as a line of best fit through the data points, is only intuitive under all-positive coding.”

2. For point 9, my understanding is that this section aims to argue for the reasonableness of the VICE assumption. However, the explanation remains unclear and unconvincing.
a. The phrase “then under VICE we can only allow the following relationship” is vague and not sufficiently explained. It would help if the authors could explicitly define why it follows logically from the VICE assumption.

The wording was inaccurate as the relationship does not follow from VICE, but the conclusion does. Thank you for bringing this to our notice. We have reworded as follows

“If, however, $\alpha$ and $\gamma$ are not assumed to be independent, we can only allow the following relationship:

$$\alpha=\frac{\left( \alpha\gamma\right)}{\sqrt{\left( \gamma^{2} \right)}}=\frac{\alpha_{0}}{\sqrt{\left( \gamma^{2} \right)}}+\frac{\left( \alpha\gamma-\alpha_{0} \right)}{\sqrt{\left( \gamma^{2} \right)}}$$

Under VICE, $\alpha\gamma$ and $\alpha_{0}$ are independent of $\gamma^{2}$. The first term therefore implies that $E\left( \alpha\right)$ is proportional to $\gamma^{-1}$ and the second that $\alpha$ varies stochastically with $\gamma^{-1}$.”

Together with the example in fig 5(d), we hope that this clarifies the implied relationship between $\alpha$ and $\gamma$ under VICE.

b. The explanations of inverse proportional to gamma, and of “stronger effect on exposure”, are difficult to follow and not strong enough. It would be great if the authors can provide clearer and stronger argument in this part, since I believe arguing the reasonableness of the VICE assumption is very important in this paper. Since the whole paper is built upon this assumption, the argumentation here needs to be much clearer and more rigorous.

We are not claiming that the precise relationship implied by VICE exists in reality, only that it is a reasonable model of it, and potentially a better fit to the data than InSIDE, as shown in the data examples. Therefore we do not attempt a rigorous defence of VICE; we only “suggest that MR-GRIP is compatible with a plausible biological model”. This is a claim that could be falsified if future analyses with MR-GRIP give implausible results. We hope that the revised text for point 2a clarifies the intuitive argument.

## Reviewer 3

While the estimation framework is clearly presented, the authors did not provide an explicit formula for the variance of the MR-GRIP estimator. This seems straightforward to derive, but it would be important to include it for completeness, since variance estimation matters in practice for inference and implementation. And it can make the method more defensible.

The causal estimate and its standard error are obtained from standard weighted least-squares theory. We have added these expressions to the Supporting Material, with a note on page 11.

Although the intercept is treated as a nuisance parameter, the paper itself notes that its p-values are highly correlated with those of MR-Egger in the simulation. It would be interesting and informative to include simulation results illustrating the performance of the intercept test under MR-GRIP, even briefly, to support this point.

We have added new tables 3 and S8 giving power for both methods, and the correlation between their P-values, in each scenario. (The correlations are lower than previously stated as we have now used Spearman correlations).
